# Burnout and moral injuries after foreign deployment among medical personnel of the German armed forces: a pre-post study

**DOI:** 10.3389/fpsyt.2024.1408849

**Published:** 2024-09-10

**Authors:** Franziska Langner, Anna Katharina Börke, Patric Muschner, Maria Muther, Andreas Reichelt, Gerd-Dieter Willmund, Ulrich Wesemann, Peter Lutz Zimmermann, Isabel Schönsee

**Affiliations:** ^1^ Bundeswehr Center for Military Mental Health, Military Hospital Berlin, Berlin, Germany; ^2^ Department of Marriage, Family And Life Councelling, Diocese Augsburg, Augsburg, Germany; ^3^ Department of Occupational Medicine, Sanitätsunterstützungszentrum Berlin, Berlin, Germany

**Keywords:** burnout, values, soldiers, military, moral injury, deployment, medical service

## Abstract

**Introduction:**

Given a high amount of workplace stressors, burnout syndrome, as a depression-related syndrome, is highly relevant for medical service soldiers. This study aims to examine their effects with regard to moral injuries and personal values following foreign deployment.

**Materials and methods:**

This longitudinal study included 91 soldiers of the German Armed Forces Medical Service. Participants completed the Maslach Burnout Inventory (MBI) and the Portrait-Value-Questionnaire (PVQ) before and after a foreign deployment as well as the Moral Injury Scale (SMBE) after deployment. Analysis has been conducted using *t*-tests to assess potential changes in MBI and PVQ scales between pre-test - t_1_ (2-4 weeks before deployment) and post-test – t_2_ (up to 6 months after deployment). In addition, correlations were examined between moral injuries (MI) after deployment and MBI scores at t_1_ and t_2_ as well as between personal values (PVQ t_1_) and MBI scores at t_1_ and t_2_.

**Results:**

The MBI subscales showed mild to moderate burnout symptoms at both pre- and post-tests, with a slight deterioration during the study period, albeit not significant. There were no significant mean differences in PVQ between measurement points. Nevertheless, PVQ self-direction and tradition at t_1_ correlated negatively with MBI INV at t_2_ (PVQ SD *r* = -.21, *p* = .043) and MBI PA at t_2_ (PVQ TR *r* = -.23, *p* = .027). Furthermore, the subscale PVQ power at t_1_ correlated positively with MBI PA at t_2_ (PVQ PO *r* = .28, *p* = .006), meanwhile PVQ universalism at t_1_ correlated positively with MBI INV at t_1_ (PVQ UN *r* = .25, *p* = .018). Furthermore, positive correlations were found between moral injuries at t_2_ (SMBE total score, SMBE_Sub1, SMBE_Sub2) and MBI subscales Emotional Exhaustion (EE; *r* = -.54, *p* = .001), Depersonalization (DP; *r* = .38, *p* = .001), and Involvement (INV; *r* = .30, *p* = .004) before and after the deployment period. No correlation was found between MI and MBI subscale Personal Accomplishment (PA).

**Conclusion:**

The results indicate that medical service soldiers exhibit mild to moderate burnout symptoms even before deployment. Significant associations between moral injuries and burnout were found in 3 out of 4 MBI subscales (EE, DP, INV). There was a significant association with a stronger moral injury and higher burnout levels, persisting both before and after the study period. Furthermore, our results suggest that personal value orientations might be meaningful predictors of burnout. Hence, causal questions regarding general work stress among medical service soldiers should be further explored in more detailed studies. Further research could lay the foundation for future approaches in psychotherapy as well as primary and secondary prevention in this field.

## Introduction

For over 20 years, Germany has witnessed an increase in burnout cases (1, 2). A meta-analysis (3) on the burnout syndrome revealed a reciprocal relationship between work-related stress and burnout. Burnout syndrome is characterized by three dimensions: feelings of energy depletion or exhaustion, increased mental distance from one’s job or work-related feelings of negativism or cynicism, as well as reduced professional efficacy. While commonly associated with mental disorders, burnout syndrome is not recognized as a distinct mental illness in the ICD classification of mental disorders. However, it can be coded as a comorbid feature in relation in coping with life’s challenges (4, 5). Due to heterogeneous definition criteria for burnout syndrome, quantifying the overall prevalence and delineating it in terms of differential diagnosis is difficult (6, 7). Meta-analyses have shown significant associations between exhaustion components of burnout syndrome (8) and depression (r = 0.520, SE = 0.012, 95% CI = 0.492, 0.547) as well as between burnout syndrome and anxiety (9) (r = 0.460, SE = 0.014, 95% CI = 0.421, 0.497). In contrast to the solely work-related genesis of burnout syndrome, studies such as those by Khammissa (10) suggest that both exogenous work-related and endogenous personal factors determine the extent and severity of symptoms.

Various studies on the prevalence of burnout syndrome in specific occupational groups revealed that professions with helping and counseling attitudes, such as healthcare, nursing or in the pedagogical field, are often affected more than other professional groups (11–13). Medical personnel play a distinct role, as workload and burnout syndrome as well as burnout syndrome and medical error rates (14) correlate positively among physicians in general (15) and emergency physicians in particular (14, 16–18).

In civilian cohorts of this occupational group, the emergence of psychological distress has been associated with workplace conditions such as experienced violence (19), the characteristics of critical events (20, 21), including ethical considerations (22), psychosocial factors (23), and the presence of resources (24). Military medical personnel may face additional stressors, such as experiencing personal threat or combat exposure during overseas deployment, which in turn may increase the risk for ill health (25, 26) and burnout (13). Consequently, studying burnout and mental health within military contexts becomes increasingly relevant.

### Burnout syndrome and mental disorders in the military

The prevalence of burnout syndrome among military personnel seems slightly lower than in society at large (11). Nevertheless, in line with findings from civilian studies (27, 28), certain military specializations show an increased likelihood of burnout syndrome (29), including personnel in operational roles, flight crews, and medical personnel, particularly in intensive care. Already in 2011, Eisenlohr et al. (6) highlighted the significance of burnout syndrome within the general medical care setting of the German Armed Forces, pointing out specific challenges in the military work environment. Differences are also reflected in specialties of military health care workers, with emergency physicians exhibiting higher levels of burnout than orthopedic surgeons (30).

Other studies have emphasized overseas deployment as a specific risk factor regarding burnout symptomatology. For instance, a study of US military personnel reported a positive correlation between the number of completed overseas deployments and an increased risk of burnout syndrome (31). Adler et al. (13) demonstrated a correlation between the perception of occupational stress among military medical personnel deployed overseas and burnout levels.

Overseas deployments put physical and psychological strains on soldiers. Comparing German soldiers with and without deployments abroad in 2012, the 12-month prevalence of PTSD increased from 0.3% to nearly 3%, with an estimated number of unreported cases of about 50% (32). The prevalence of mental disorders among deployed participants in a study of the German Armed Forces was 21.4% (33, 34). Previous studies have shown that the 12-month prevalence of mental disorders in German soldiers without deployment history is comparable to that of the general population (35). Research indicates that it is not the deployment itself, but instead high combat exposure with threats to life and physical integrity that is linked with increased prevalence rates of anxiety disorders and PTSD (35, 36). An international study demonstrated varying prevalence rates depending on the sending country and the deployment region (36). The stressors faced by soldiers during overseas deployments are likely to favor the phenomenon of burnout syndrome. Further insights could be provided by studies examining emotional stress (37) and the development of depressive disorders and anxiety disorders (20, 38) during deployment, as these show similarities with the symptoms of burnout syndrome (39, 40).

### Moral injury in the context of foreign deployment

In addition, soldiers on deployment may face traumatic experiences that challenge their individualized psychological value systems. These conflicts often arise from the disparity between internalized Western-liberal values and prevailing views in the country of deployment. Litz et al. (41) have introduced the concept of “moral injury” (MI), in which soldiers are morally burdened by experiences during deployment, either by their own offenses (e.g. exercising or witnessing disproportionate violence), or when they themselves do not act or are not allowed to act (“leadership decisions that were life-ending”, “neglect”). Depending on the type (42) of PMIES (stemming from the actions of self and other), Dale et al. (43) showed an increased risk of specific mental illnesses (self MI: more burnout syndromes/depression, other MI: more anxiety).

Other authors define moral injury as betrayal by authority figures in extreme situations (44), actions that contradict one’s own moral convictions (45) or as breaks in the moral self-concept that cause intense emotional reactions such as guilt and shame (46). Such experiences can have a profound impact on psychological well-being and the integrity of moral character (47). Moral character is seen as dynamic and malleable, influenced by personal experiences, cultural norms and social interactions (48). Moral dissonances, which arises when a person performs actions or witnesses’ events that contradict their moral beliefs (47), can undermine confidence in one’s own moral judgment and sow doubt in one’s own moral integrity.

The consequences of MI are negative feelings and cognitive dissonance. If these cannot be reduced, they can lead to secondary mental illnesses, accompanied by feelings of guilt and shame (41, 49). Studies (48, 50–52) show that MI, like the confrontation with adversity and the killing of opponents, increase the frequency of mental illness after deployment. The moral fragmentation that those affected experience when their actions and beliefs do not coincide also leads to an incoherent perception of their own moral principles (48). In addition, MI represents a differentially mediating variable between the stressor “confrontation with hardship, suffering, violence in the population” and the psychological syndromes “PTSD” and “depression” on the one hand and “burnout syndrome” and alcohol abuse on the other (53, 54).

Moral injury and burnout are two different but often co-occurring phenomena that occur in the context of occupational stress (41). Both can have serious psychological and physical consequences for those affected, but their causes, manifestations and effects differ considerably. In the military context, moral injury results from acts of violence or witnessing civilian casualties, leading to moral dilemmas (42, 48) or from situations in which medical personnel have to deal with end-of-life decisions or medical errors (55, 56). Burnout, on the other hand, is caused by chronic professional overload and emotional stress, e.g. intense physical and psychological demands as well as traumatic events lead to burnout (57) or the emotional strain of dealing with seriously ill patients by medical staff (58, 59). So far it is unknown, whether moral injury leads to higher or lower levels of burnout. For the first time, this research paper addresses this question in order to provide some preliminary results on this topic.

### Personal values as influencing factors of mental health

In the medical context (medical service), unresolvable feelings of guilt, shame and self-blame (e.g. due to limited medical resources for the further treatment of locals in the war zone), the experience of potentially morally injurious events (PMIE) and resulting conflicts between personal and professional values may increase the risk for mental illness (53, 60). Furthermore, initial research suggests that values may also play an important role in predicting levels of burnout and engagement (61) as well as mental health (62). From a theoretical perspective, values have also been integrated into the areas of worklife model of burnout as one of six key areas in which a person-job imbalance might affect an individual’s level of experienced burnout (7, 61, 63). It therefore seems necessary to examine personal value orientations in relation to mental health and burnout more closely.

Values, as central beliefs that guide behavior and attitudes, have been identified in several studies (50, 64–66) as personality-related influencing factors in the development and progress of mental health (65). Long-term studies and cognitive consistency theories demonstrate the relative stability (Bardi 2003) of values in adulthood. Biological and developmental psychology perspectives (67, 68) complement this view by examining the role of socialization and genetic factors in the formation of values. Schwartz (69, 70). identified ten types of values (see **Table 1**), which represent different motivational goals deriving from overarching universal human needs. An extensive overview of the relationship between value orientations and mental health is given by Heim et al. (71).

**Table 1:** Definitions of ten value constructs in terms of their goals (70).

Value	Definition
*Power*	Social status and prestige, control or dominance over people and resources.
*Achievement*	Personal success through demonstrating competence according to social standards.
*Hedonism*	Pleasure and sensuous gratification for oneself.
*Stimulation*	Excitement, novelty, and challenge in life.
*Self-direction*	Independent thought and action-choosing, creating, exploring.
*Universalism*	Understanding, appreciation, tolerance and protection for the welfare of all people and for nature.
*Benevolence*	Preservation and enhancement of the welfare of people with whom one is in frequent personal contact.
*Tradition*	Respect, commitment and acceptance of the customs and ideas that traditional culture or religion provide the self.
*Conformity*	Restraint of actions, inclinations, and impulses likely to upset or harm others and violate social expectations or norms.
*Security*	Safety, harmony and stability of society, of relationships, and of self.

Meanwhile the majority of previous research demonstrates the stability of personal value orientations (72–75), more recent research, such as that by Bardi et al. (73), challenges the theory of value stability and shows that specific events such as the COVID-19 pandemic or terroristic attacks can cause temporary changes in values. The studies by Bojanowska et al. (76) and Daniel et al. (77) suggest that exceptional circumstances such as a pandemic can lead to temporary adjustments in values that do not necessarily reflect long-term changes.

The distinction between temporary and stable changes in values requires a differentiated view of time perspectives and contexts. In the context of foreign assignments, stressful experiences can trigger temporary changes in values by stimulating a reflection or reassessment of life goals and convictions (78, 79) or by raising existential questions. In addition, it seems that people under stress can temporarily adapt their values in order to cope better with challenges (80). Nevertheless, studies show that fundamental values remain stable over longer periods of time and can serve as orientation systems and sources of resilience in stressful situations (81, 82).

So far, we only identified one study reporting on personal values and mental health in the military context (83). Here, certain value types have been identified as commonly held by soldiers, such as benevolence and universalism. These seem to support moral professional conduct but also make individuals more susceptible to moral conflicts. Conversely, individuals who are more materially oriented and less compassionate (high hedonism) may be protected from such stressors. Further evidence in this field of research is missing. Hence, in order to address this research gap, we examine personal values in the military context looking more closely at their association with burnout and their stability over time.

### Objectives and hypotheses

To date, studies on the development of burnout syndrome among medical personnel in the German Armed Forces considering personal value orientations and moral injuries in overseas deployments are missing. It seems that value orientations and moral injuries influence the pathogenesis of mental illnesses and are influenced by participation in military deployments (50, 66). Overall, our study underscores the importance of understanding burnout syndrome, particularly in military contexts, and highlights the need to examine how value change and how MI contribute to soldiers’ burnout symptoms after deployment.

This project provides initial results on the relationship between burnout symptoms, value orientations, and moral injury among medical personnel of the German Armed Forces deployed in overseas missions. We hypothesize that 1) there will be an increase in burnout symptomatology after the deployment period, 2) personal values are associated with burnout and remain stable even after a six months period after deployment and 3) a stronger experience of moral injury is associated with significantly higher levels of burnout.

## Materials and methods

### Sample

In this quantitative study, 91 medical soldiers were surveyed via structured questionnaires between December 2014 and November 2016. The study population represents the official proportion of female soldiers in the German medical service of 44.6% and was not specifically selected.

Participants were recruited during a routine health examination (BA90/5) preceding their deployment to various missions abroad (e.g., Afghanistan, Mali, Kosovo). Participation in the study was optional, with interested individuals being briefed about the research project during individual consultations with study personnel. Participants were required to have a minimum deployment duration of 30 days and meet the age criteria of 20-60 years. Exclusion and discontinuation criteria included acute mental illness, suicidality, or concurrent participation in another study.

### Research procedure

The study was conducted in accordance with the Declaration of Helsinki guidelines. A positive ethics vote from the Humboldt University of Berlin (Charité) was obtained (No. EA1/203/13). All patients gave informed written consent for an examination and voluntarily participated in the study. The study was registered by the Bundeswehr Medical Academy under a special research number 07-K4-S321315. There was no third-party funding.

The participants were examined at two times of measurement. The pretest (t_1_) was conducted 2-4 weeks before deployment (Maslach Burnout Inventory + Portrait Value Questionnaire). The posttest (t_2_) was conducted up to 6 months after the end of the deployment (Maslach Burnout Inventory + Portrait Value Questionnaire + Moral Injury Event Scale). Immediate post-measurement was omitted due to difficult scheduling of out-of-mission dates, frequent subsequent vacation and rest days, and other personal reasons of the participants.

### Measures

#### Maslach burnout inventory

The Maslach Burnout Inventory (MBI) in its German version (84) consists of 25 items comprising four subscales: emotional exhaustion (EE; 9 items), depersonalization (DP; 5 items), personal accomplishment (PA; 8 items), and involvement (INV; 3 items). Participants rated themselves on a seven-point intensity scale ranging from 0 (= never) to 6 (= daily). Whereas the subscales EE, DP and PA showed acceptable inter-item correlations of *α* = .88, *α* = .82 and *α* = .72, respectively, the inter-item correlation of the subscale INV was *α* = .53. Despite the low inter-item correlation, INV was also surveyed in order to check whether it is particularly important for military medical personnel. For example, a high level of involvement could indicate a lower capacity for self-detachment and thus represent a stressor that has a negative effect on the individual’s energy balance and a positive effect on increasing burnout. Means and standard deviation for MBI-HSS scales can differ among different occupational groups. Maslach (85), for example, reports on a group of medical personnel (*N* = 1104, MBI EE *M* = 22.19, *SD* = 9.53; MBI DP *M* = 7.12, *SD* = 5.22, MBI PA *M* = 36.53, *SD* = 7.34) whose standard values differ from Mental health personal (*N* = 730, MBI EE *M* = 16.89, *SD* = 8.90; MBI DP *M* = 5.72, *SD* = 4.62, MBI PA *M* = 30.87, *SD* = 6.37).

The MBI manual (63) defined burnout (BO) cutoff values for each item with “low”, “medium” and “high” levels of burnout (MBI EE low <16, med = 18-29, high >30; MBI DP low <5, med 6-7-, high >12; MBI PA low <33, med 34-39, high >40) by dividing the normative sample into three equally-sized groups of 33.3%. A differentiation is made between the normative data of medical personnel (*N* = 1104, MBI EE *M* = 22.19, *SD* = 9.53; MBI DP *M* = 7.12, *SD* = 5.22, MBI PA *M* = 36.53, *SD* = 7.34) and mental health personnel (*N* = 730, MBI EE *M* = 16.89, *SD* = 8.90; MBI DP *M* = 5.72, *SD* = 4.62, MBI PA *M* = 30.87, *SD* = 6.37). The lack of external validation and the rather arbitrary test construction prevent these cutoff values and severity classifications from constituting diagnostically valid instruments for medical practice. Therefor the cut-off scores were removed in the latest version of MBI-HSS MP. For research purposes, the values of the individual scales continue to be used to assess the BO level: higher values of the EE, DP and INV scales indicate a higher degree of burnout, lower values of the PA scale indicate a higher degree of BO (86, 87).

#### Portrait value questionnaire

The Portrait Value Questionnaire (PVQ) comprises 40 items (69, 88) and assesses the expression of ten value types (e.g., power, achievement, security). Participants rate themselves on a 6-point Likert scale (1 = very dissimilar; 6 = very similar). Internal consistency ranges from *α* = .35–.83, and test-retest reliability (82) ranges from.62 to.82.

#### Moral injury event scale

The Moral Injury Event Scale (SMBE) includes 9 items and assesses the burden on participants caused by events that violate moral beliefs and values via a 6-point Likert scale (1 = strongly disagree, 6 = strongly agree). Item responses were averaged, such that higher scores on the SMBE correspond to a greater intensity of exposure and distress (49). In the German version, internal consistencies for moral injury caused by others (SUB1, 5 items) and for betrayal of moral standards (50) due to one’s own misconduct (SUB 2, 4 items) ranged from *α* = .82 to *α* = .78, respectively.

### Statistics

Data analysis was conducted using IBM Statistics SPSS 25. Initially, a descriptive analysis of the sample was performed based on sociodemographic information and the assessment of burnout symptoms. To examine whether differences occurred over time, a paired sample *t*-test was used. The requirements for the *t*-test were met. A test for normal distribution was omitted, as simulation studies show that the *t*-test is robust to this violation (89). Furthermore, changes in value orientations over time were examined using a *t*-test for related samples.

Additionally, Pearson correlation analyses were conducted to see if there was a correlation between Moral Injury (SMBE total score, SUB 1, and SUB 2) and Burnout (EE, PA, DP, IV) subscales. The prerequisites for a correlation (interval scaled variables, linearity) were met. Outliers were left in the data set. The sample size was determined using G-Power. Method used: Correlation, Bivariate normal model. In a power analysis conducted based on a conservatively estimated Pearson correlation of *r* = 0.3 (mean effect), *alpha error* =.05 and *power* (1-beta err. prob.) = 0.80 the minimum sample size (total sample size) is *N* = 67.

## Results

### Sample description

In total, 91 medical soldiers, including male (*N*=48; 52.7%) and female (*N*=43; 47.3%) soldiers with an average age of 33.55 years (*SD* = 6.07) took part in this study. Of these, 80.3% reported being in a relationship, while 19.8% reported being single. Overall, 75.8% belonged directly to the Medical Service section of the armed service, while the remaining participants were soldiers from other military services such as the Land Forces (8.8%), the Air Force (4.4%), and the Navy (2.2%). No information regarding the specific area of service was provided by 8.8% of the participants. The various ranks, which reflect the hierarchy within the German Armed Forces, are broken down as follows, from the lowest (enlisted ranks), through the middle (non-commissioned officers) to the highest ranks (officers): 6.6% NATO rank OR-1 to OR-4, 48.4% NATO rank OR-5 to OR-9, 44% NATO rank OF-1 to OF-5 and 1.1% provided no information.

The deployment was the first overseas assignment for 45.1% of the participants. The other participants had previously participated in an average of 4.60 deployments (*SD* = 12.82; *Mode* = 1), the average duration was 238.83 days (*SD* = 204.89). In the current deployment, participants were deployed to various operational areas of the German Armed Forces (45.1% Kosovo; 36.3% Afghanistan; 9.9% Mali; 5.5% other areas; 3.3% no information provided.

### MBI

The initial mean values of the MBI (see **Table 2**) in our study sample showed deviating values in individual subscales compared to the normative samples, depending on the occupational subgroup. For example, medical personnel show lower levels in EE and DP as well as a higher level in PA than our study sample. In comparison to the subgroup of mental health personnel the reported means of EE, DP and PA are higher. Using the burnout cutoff values the MBI subscales Depersonalization (MBI_DP *M*_t1_ = 6.93) and Personal Accomplishment (MBI_PA *M*_t1_ = 36.76) indicated a moderate level of burnout at t_1_. The scales Emotional Exhaustion (MBI_EE *M*_t1_ = 17.0) and Involvement (MBI_INV *M*_t1_ = 3.72) reflected a low level of burnout.

**Table 2:** t-test for dependent samples of the MBI including means and standard deviations for MBI-HSS Scales.

Scale	*M*_t1_	*SD*	*M*_t2_	*SD*
MBI_EE	17.0	10.03	17.02	9.99
MBI_DP	6.93	6.03	6.99	5.93
MBI_PA	36.76	8.07	35.81	8.59
MBI_INV	3.72	3.00	4,22	2.91

MBI, Maslach Burnout Inventory; EE, emotional exhaustion; DP, depersonalization; PA, personal accomplishment; INV, involvement; t_1_ = measurement time point 1; t_2_ = measurement time point 2.

During the study period, a slight deterioration was observed, but paired t-test analysis showed no significant changes over time in the MBI subscales: MBI_EE *t (90*) = - 0.030, *p* = .976, *d_z_* = 0; MBI_DP *t*(90) = - 0.127, *p* = .899, *d_z_* = 0; MBI_PA *t*(90) = 1.46, *p* = .147, *d_z_* = 0.153; MBI_INV *t*(86) = -1.75, *p* = .083, *d_z_* = - 0.188.

### PVQ

Furthermore, paired *t*-tests were conducted to examine whether the values of the PVQ changed from t_1_ to t_2_ (see **Table 3**). There were no significant changes in the subscales between the two times of measurement.

**Table 3:** *t*-test for dependent samples of the centered values PVQ.

Scale	*M_t1_*	*SD_t1_*	*M_t2_*	*SD_t2_*	*t*	*df*	*p*
PVQ_Universalism	0.09	0.66	0.07	0.68	0.40	86	.688
PVQ_Benevolence	0.61	0.53	0.56	0.49	1.25	86	.213
PVQ_Conformity	-0.12	0.63	-0.15	0.70	0.66	86	.512
PVQ_Tradition	-0.91	0.71	-0.85	0.65	-1.19	86	.236
PVQ_Security	0,22	0.58	0.25	0.51	-0.57	86	.569
PVQ_Power or Force	-0.59	0.92	-0.64	0.87	0.79	86	.431
PVQ_Performance	0.09	0.81	0.13	0.77	-0.80	86	.422
PVQ_Hedonism	0.10	0.84	0.61	0.88	-0.05	86	.962
PVQ_Stimulation	-0.46	0.95	-0.45	0.86	-0.19	86	.848
PVQ_Self-direction	0.63	0.51	0.63	0.45	0.14	86	.885

PVQ, Portrait Value Questionnaire; t_1_ = measurement time point 1; t_2_ = measurement time point 2; p_(2-sided)_ <.05.

To check for an association between the PVQ and MBI subscales at t_1_ and t_2_ Pearson correlations were calculated. Only a few significant correlations were found.

PVQ Self-direction at t_1_ correlates negatively with MBI INV (see **Table 4**) at t_2_ (PVQ SD *r* = -.21, *p* = .043). The higher self-direction at t_1_, the lower the involvement in MBI scale at t_2_. Furthermore, the subscale PVQ Power at t1 correlates positively with MBI PA at t_2_ (PVQ PO *r* = .28, *p* = .006). Lower scores in the MBI INV and higher values in the MBI PA can indicate a lower level of burnout. Thus, higher values in the PVQ Self-direction and lower scores in PVQ PA at t_1_ can be associated with an increased occurrence of burnout symptoms after deployment at t_2_.

**Table 4:** Corellation matrix with scales PVQ subscales at t_1_ and MBI subscales at t_2_.

Scale		MBI_EE t_2_	MBI_DP t_2_	MBI_PA t_2_	MBI_INV t_2_
PVQ_Selfdirection t_1_	*r*	-.192	.019	-.019	-.217*
	*p*	.072	.862	.862	.043
	*N*	89	89	89	87
PVQ_Power_t_1_	*r*	-.025	.094	.287*	-.019
	*p*	.815	.382	.006	.861
	*N*	89	89	89	87
PVQ_Universalismus t_1_	*r*	-.058	-.014	-.023	.141
	*p*	.59	.896	.83	.192
	*N*	89	89	89	87
PVQ_Achiement t_1_	*r*	.187	.051	.156	.035
	*p*	.08	.638	.143	.745
	*N*	89	89	89	87
PVQ_Security t_1_	*r*	.143	.043	.015	-.121
	*p*	.182	.686	.888	.263
	*N*	89	89	89	87
PVQ_Stimulation t_1_	*r*	.017	.034	-.104	.076
	*p*	.873	.753	.33	.487
	*N*	89	89	89	87
PVQ_Conformity t_1_	*r*	.06	-.068	-.121	.055
	*p*	.576	.524	.259	.611
	*N*	89	89	89	87
PVQ_Tradition t_1_	*r*	.122	-.008	-.234*	-.111
	*p*	.253	.938	.027	.305
	*N*	89	89	89	87
PVQ_Hedonism t_1_	*r*	-.193	-.032	-.041	-.05
	*p*	.071	.768	.7	.643
	*N*	89	89	89	87
PVQ_Benevolence t_1_	*r*	-.181	-.167	.073	.132
	*p*	.089	.117	.497	.223
	*N*	89	89	89	87

t_1_ = measurement time point 1; t_2_ = measurement time point 2; r, Pearson Correlation coefficient; * Correlation is significant at the 0.05 level (2-tailed).

PVQ Tradition at t_1_ correlates negatively with MBI PA at t_2_ (PVQ TR *r* = -.23, *p* = .027) The higher tradition at t_1_, the lower the MBI PA at t_2_. There was a positive correlation found between PVQ Universalism at t_1_ and MBI INV (see **Table 5**) at t_1_ (PVQ UN *r* = .25, *p* = .018). PVQ Hedonism at t_1_ correlates negatively with MBI EE at t_1_ (PVQ HE *r* = -.26, *p* = .012). The higher hedonism at t_1_, the lower the emotional exhaustion in MBI at t_1_. In addition, PVQ Benevolence at t_1_ correlates negatively with MBI DP at t_1_ (PVQ BE *r* = -.25, *p* = .016), indicating that the higher benevolence at t_1_, the lower depersonalization in MBI at t_1_. According to Cohen (90, 91), all correlations represent a small effect size. The detailed tabular view can be viewed in the appendix.

**Table 5:** Corellation matrix with scales PVQ subscales at t_1_ and MBI subscales at t_1_.

Scale		MBI_EE t_1_	MBI_DP t_1_	MBI_PA t_1_	MBI_INV t_1_
PVQ_Selfdirection t_1_	*r*	-.09	.131	-.014	-.122
	*p*	.4	.223	.895	.262
	*N*	89	89	89	86
PVQ_Power_t_1_	*r*	.031	.097	.112	-.073
	*p*	.772	.368	.296	.502
	*N*	89	89	89	86
PVQ_Universalismus t_1_	*r*	-.021	-.037	.052	.255*
	*p*	.843	.732	.626	.018
	*N*	89	89	89	86
PVQ_Achiement t_1_	*r*	.16	.051	-.001	-.179
	*p*	.134	.636	.989	.099
	*N*	89	89	89	86
PVQ_Security t_1_	*r*	.103	.066	-.05	-.155
	*p*	.337	.541	.644	.153
	*N*	89	89	89	86
PVQ_Stimulation t_1_	*r*	.033	.111	-.132	0
	*p*	.759	.301	.219	1
	*N*	89	89	89	86
PVQ_Conformity t_1_	*r*	.001	-.157	-.025	.137
	*P*	.995	.143	.817	.207
	*N*	89	89	89	86
PVQ_Tradition t_1_	*r*	.049	.008	-.139	.051
	*p*	.649	.939	.192	.64
	*N*	89	89	89	86
PVQ_Hedonism t_1_	*r*	-.264*	-.062	.038	-.123
	*p*	.012	.561	.726	.259
	*N*	89	89	89	86
PVQ_Benevolence t_1_	*r*	-.094	-.254*	.189	.142
	*p*	.379	.016	.075	.191
	*N*	89	89	89	86

t_1_ = measurement time point 1; r, Pearson Correlation coefficient; * Correlation is significant at the 0.05 level (2-tailed).

### SMBE

**Table 6** shows the moral injuries experienced by soldiers after deployment (t_2_). Compared to a possible total score of 54 points, the overall mean score of SMBE (*SMBE in total*) indicates a rather low score (*M*_t2_ = 21.79), suggesting a lower level of distress in general. Examining the individual subscales, it can be highlighted that soldiers experienced lower moral injury from their own actions (SMBE_Sub2 *M*_t2_ = 7.49) compared to the actions of others (SMBE_Sub1 *M*_t2_ = 14.29).

**Table 6:** Descriptive statistic.

Scale	*M*_t2_	*SD*	*N*
SMBE_Sub1	14.29	5.67	91
SMBE_Sub2	7.49	4.40	91
SMBE_in total	21.79	8.69	91

SMBE, Moral Injury Scale; SUB1 = caused by others actions; SUB2 = caused by own actions.

**Table 7** demonstrates that moral injuries resulting from the actions of others as well as from one’s own actions positively correlate with the Emotional Exhaustion (EE) scale of the MBI at t_1_ (SMBE_Sub1 *r* = .48, *p* = .001, SMBE_Sub2 *r* = .45, *p* = .001, SMBE_Sub in total *r* = -.54, *p* = .001) and t_2_ (SMBE_Sub1 *r* = .46, *p* = .001, SMBE_Sub2 *r* = .38, *p* = .001, SMBE_Sub in total *r* = .49, = .001). According to Cohen (92), this represents a medium effect size. The higher the experienced number of moral injuries caused by the actions of others or by one’s own actions, the higher the emotional exhaustion. These findings are reflected in conjunction with the Depersonalization (DP) scale. There is also a medium positive correlation with moral injuries caused by others (SMBE_Sub 1 *r* = .33, *p* = .001) as well as moral injuries caused by one’s own actions (SMBE_Sub 2 *r* = .32, *p* = .002) and total moral injuries (SMBE_Sub in total *r* = .38, *p* = .001) at t_1_. At t_2_, the correlation changes to weakly positive for moral injuries caused by others and by one’s own actions (SMBE_Sub1 *r* = .26, *p* = .011, SMBE_Sub2 *r* = .28, *p* = .007), while the total score of SMBE_Sub in total continues to show a medium positive correlation (SMBE_Sub in total *r* = .31, *p* = .002). Overall, this means that the more moral injuries a soldier experiences, the stronger the experience of depersonalization.

**Table 7:** Correlation matrix with scales MBI and SMBE.

Scale	SMBE_Sub 1			SMBE_SUB 2			SMBE_SUB in total		
	*r*	*p*	*N*	*r*	*p*	*N*	*r*	*p*	*N*
*MBI_EE _t1_*	.48	.001	91	.45	.001	91	.54	.001	91
*MBI_DP _t1_*	.33	.001	91	.32	.002	91	.38	.001	91
*MBI_PA _t1_*	-.09	.380	91	-.10	.340	91	-.11	.290	91
*MBI_INV _t1_*	.20	.059	87	.34	.001	87	.30	.004	87
*MBI_EE _t2_*	.46	.001	91	.38	.001	91	.49	.001	91
*MBI_DP _t2_*	.26	.011	91	.28	.007	91	.31	.002	91
*MBI_PA _t2_*	-.10	.327	91	-.15	.165	91	-.14	.179	91
*MBI_INV _t2_*	.22	.034	88	.26	.013	88	.28	.008	88

SMBE, Moral Injury Scale; SUB1 = caused by own actions; SUB2 = caused by others; t_2_ = measurement time point 2.

Furthermore, the subscale Involvement (INV) of the MBI at t_1_ shows a medium positive correlation with moral injuries caused by one’s own actions as well as with the total score of SMBE (SMBE_Sub 2 *r* = 0.34, *p* = .001, SMBE_Sub in total *r* = .30, *p* = .004). No significant relationship could be found for moral injuries caused by others and INV. At t_2_, both subscales of SMBE show a low positive correlation with INV (SMBE_Sub1 *r* = .22, *p* = .034, SMBE_Sub2 *r* = .26, *p* = .013). The total score of SMBE shows a medium positive correlation (Sub in total *r* = .28, *p* = .008) with INV. Again, it is evident that the higher the experienced moral injuries caused by one’s own actions or by the actions of others, the higher the experience of involvement in the MBI. No significant relationships could be found between moral injuries and the Personal Accomplishment scale of the MBI.

## Discussion

For the first time, this study examined the relationship between burnout syndrome, value orientations, and moral injuries among medical service soldiers after participating in a foreign mission.

The psychological strain and the increased incidence of mental illnesses among soldiers participating in overseas deployments have been demonstrated in various studies (33, 75). Additionally, Adler (13) demonstrated significant positive correlations between the perception of occupational stressors and the degree of burnout among medical personnel in the US military. Zimmermann et al. also found a significant increase in depression over the course of deployment among soldiers without a medical background (64). In line with these findings and taking into account the similar symptom expression between burnout syndrome and depression (5, 8), we expected to see an increase in burnout symptomatology among medical service soldiers after foreign deployment.

Comparing the results of the MBI from t_1_ to t_2,_ it became evident that medical soldiers already showed mild to moderate burnout symptoms in the subscales of depersonalization and personal accomplishment before deployment. These slightly worsened during the deployment, but contrary to our expectations this change wasn’t significant. Thus, we reject our first hypothesis. In interpreting this finding, one must consider the chosen normative sample. Based on normative samples with medical personnel, we saw a comparatively low burnout burden among the medical soldiers in our study. However, compared to the normative sample group of mental health professionals, higher values were observed in EE and DP (85). However, the normative samples did not account for adjusted values such as age and gender, which might explain some of the differences between the amount of burnout symptoms in our study sample and that of the normative sample group.

Furthermore, in contrast to Houkes and Watson (15), our study did not exclusively survey medical personnel, so not all medical soldiers may have been exposed to the same stressors. Overall, 75% of the participants were members of the medical service, the remaining participants belonged to other branches of the armed forces (Navy, Army, Air Force). Nevertheless, this doesn’t prevent them from working in the medical service. Further inferences about medical duties can be made by looking at the rank groups, with the group of officers, comprising 44%, most likely being deployed as medical officers. Our study replicates the results of an American study, which also found no increase in burnout syndrome among soldiers deployed in Iraq (93). Overall, this indicates that further research is needed in this area, as also described by Chambel et al. (94).

As personal values might be involved in the development of burnout symptomatology, we looked closer at the concept of personal values and their possible association with burnout. Firstly, we hypothesized that personal values remain stable over time. By assessing the changes in the PVQ from t1 to t2, no significant differences could be found. This confirmed our hypothesis and seems to support the idea that values are similar to personality factors in influencing behavior over long periods of time (95). Nevertheless, because of the high variability in measuring time points after deployment (t_2_) value changes might not have been detected. On the one hand, these could result from contextual factors, such as potential stressors following repatriation (relationship issues, reintegration into domestic service, and challenges in finding meaning in domestic activities). On the other hand, the process of value adaptation might follow a reflection process, which hasn’t occurred yet when the data collection, as in our study, happens relatively early post-deployment. Addressing these contextual and timely factors would be a valuable addition to future studies.

Regarding the association between values and burnout, this paper can only provide some initial results. Since very few studies have addressed this research topic, no specific hypothesis about the direction of the association nor the most relevant type of values could be defined beforehand. Nevertheless, our findings suggest that a higher expression in the values of self-direction and power before deployment is associated with a lower value in the burnout subscale of involvement and a higher value in the subscale of personal accomplishment after deployment, respectively. Cautiously said, the more one is able to establish and develop one’s own life path (value of self-direction) and the more power one feels over other people and resources (value of power) before deployment, the lower the level of burnout after deployment.

In contrast, a higher expression in the value of tradition seems to be associated with a lower value in the burnout subscale of personal accomplishment. This finding suggests that the more one holds on to traditional customs or norms of behavior (value of tradition) before deployment, the higher the level of burnout after deployment.

Interestingly, certain values at t_1_ were correlated with some of the burnout subscales before deployment only. For example, the value of universalism (being interested in protecting the well-being of people or the environment) was positively correlated with a higher score in the subscale of involvement (pointing towards a higher level of burnout) prior to being deployed. Meanwhile, enjoying life’s goals and events (value of hedonism) and aiding people with whom one is in close contact (value of benevolence) seems to be correlated negatively with the subscale of emotional exhaustion and depersonalization, respectively.

To our knowledge, the only study reporting on personal value orientations and psychiatric symptoms within the military has been conducted by Zimmermann et al. (72). The authors found the values of power and hedonism to be negatively correlated with the probability of PTSD, meanwhile the values of universalism and tradition increased the probability of developing PTSD after foreign deployment. Furthermore, Maercker et al. (62) identified the value of self-direction as a predictor of resilience, suggesting that personal value orientations are meaningful predictors of mental health.

Our findings seem to point into a similar direction. However, as they are only based on simple correlations and were not hypothesis-driven, the interpretation of the results must be viewed with caution. Nevertheless, we believe that these initial findings should be further investigated in follow-up studies in order to better understand the predictive potential of personal values on mental health.

In our third hypothesis, we expected that increased experiences of moral injuries during deployments would be significantly associated with elevated burnout levels. This hypothesis was partly confirmed as significant correlations were found between moral injuries and three of the four burnout subscales (Emotional Exhaustion, Depersonalization, and Involvement).

Interestingly, these correlations were found before and after deployment, suggesting two different interpretations. First, one could argue that early burnout levels pre deployment influence the experience of moral injuries during deployment. For instance, burnout symptoms such as emotional exhaustion might make soldiers more susceptible to morally questionable behavior. Second, experiencing moral injuries seems to be associated with elevated burnout levels after deployment. This interpretation could be seen in line with the theoretical model of the areas of worklife model of burnout which proposes that the greater the perceived incongruity between one’s own ideals and motivations the greater the likelihood of burnout (61).

Overall, since simple correlations are unable to allow for causality, further research is necessary to better understand the association between moral injury and burnout.

Furthermore, medical soldiers in our study sample reported a low incidence of moral injuries in the SMBE total score. The frequency of moral injuries caused by others was higher than that by their own actions. A similar distribution was observed in a non-military study of medical personnel during the COVID-19 pandemic (43), emphasizing the influence of self-induced moral injuries (Self MI) on burnout syndrome. The overall low symptom burden can be seen in line with previous findings by Wittchen et al. (32). Here, the frequency of stressful events during overseas deployments was significantly lower for medical soldiers compared to combat troops. The nature of the deployment (combat mission, security detail, and associated treatment of civilians in medical facilities with available resources) could also influence the confrontation with potentially stressful and morally significant events. In addition, the German Armed Forces have various regulations within deployment contingents. For instance, the surgical treatment of civilians being transferred to local facilities post-treatment, varies greatly, potentially leading to various moral burdens across different deployment contingents (96). These detailed correlations warrant further investigation in subsequent studies. The association between moral injury at the end of deployment and burnout was observed both before and after the examined deployment interval. Since in this study moral injuries were measured only at the end (t_2_) of the deployment, a specific attribution to the deployment experiences is therefore only partially feasible.

For the first time, our results demonstrate an association between moral injuries and the level of burnout symptoms in medical soldiers. Additionally, a high pre-existing burden might be considered a risk factor for developing burnout syndrome post-deployment. Moreover, personal value types might be meaningful predictors of burnout. These preliminary findings should be further investigated in future controlled studies to identify additional influencing factors. Although soldiers undergo medical pre-screening before deployment to determine their fitness for duty, replicating our results could prompt consideration of screening measures for psychological symptoms to aid evaluating physicians. Moreover, leadership support for medical personnel during extremely stressful work experiences, such as the COVID-19 pandemic, was identified as a protective factor by Dale (43). Therefore, strengthening personal resilience and robustness (97) and destigmatizing mental health issues through education on moral injuries and burnout syndrome (98) could be beneficial topics in leadership training within the German Armed Forces.

## Limitations of the study

This study has several limitations, including the lack of randomization and the absence of a control group, which limits the comparability of burnout development and value changes with non-deployed soldiers. Additionally, changes over time cannot be ruled out.

In a participant group comprised solely of medical service soldiers, it is not possible to clearly distinguish general soldier burdens from those specific to the medical service. Thus, the effects found should only be considered in the context of medical soldiers deployed on overseas missions. The sample size is relatively small, which might explain the lack of significant results due to underpowering and overestimated effect sizes. The sample also exhibits high heterogeneity; while the gender distribution is balanced within the medical service of the German armed forces, women are significantly underrepresented in the entire Bundeswehr (13% in 2022).

Another limitation arises from the unclear causal mechanisms and the fact that the majority of deployment participants (54.9%) had multiple overseas deployments, thus accumulating deployment experiences. The chosen test method, which generally inquires about the burden of past moral injuries without specifying a particular time frame, makes the observed relationship with burnout symptoms non-specific. Furthermore, moral injuries acquired domestically during general service as medical soldiers are also to be expected, as demonstrated in studies on military (30) and non-military medical personnel (27, 28).

It is plausible that the delayed timing of the surveys at *t*2 might also reflect general burdens such as family environment or reorientation to domestic life for the soldiers. Future studies should either separately inquire about these factors or conduct the second measurement immediately after return from deployment. Potential alternative causes for the altered response behavior could also be attributable to other unobserved factors, such as age, rank, responsibility, occupational role, type of event, and prior experiences (99, 100).

## Data Availability

The original contributions presented in the study are included in the article/supplementary material. Further inquiries can be directed to the corresponding author.
